# Researching the future: scenarios to explore the future of human genome editing

**DOI:** 10.1186/s12910-023-00951-8

**Published:** 2023-09-21

**Authors:** Cynthia Selin, Lauren Lambert, Stephanie Morain, John P. Nelson, Dorit Barlevy, Mahmud Farooque, Haley Manley, Christopher T. Scott

**Affiliations:** 1 School for the Future of Innovation in Society at Arizona State University, PO Box 876002, 85287-6002 Tempe, AZ USA; 2 School of Sustainability at Arizona State University, 4th floor, Walton Center for Planetary Health, 85281 Tempe, AZ USA; 3 Berman Institute of Bioethics, Johns Hopkins University, 1809 Ashland Ave, 21212 Baltimore, MD USA; 4 School of Public Policy, Georgia Institute of Technology, 685 Cherry St., Suite 107, 30332 Atlanta, GA USA; 5 Center for Medical Ethics and Health Policy, Baylor College of Medicine, One Baylor Plaza, Suite 310D, 77030 Houston, TX USA; 6 Consortium for Science, Policy and Outcomes, Arizona State University, 1800 I Street, 20006 Washington, DC USA

**Keywords:** Anticipation, Human genome editing, Scenario planning, Governance, Bioethics

## Abstract

**Background:**

Forward-looking, democratically oriented governance is needed to ensure that human genome editing serves rather than undercuts public values. Scientific, policy, and ethics communities have recognized this necessity but have demonstrated limited understanding of how to fulfill it. The field of bioethics has long attempted to grapple with the unintended consequences of emerging technologies, but too often such foresight has lacked adequate scientific grounding, overemphasized regulation to the exclusion of examining underlying values, and failed to adequately engage the public.

**Methods:**

This research investigates the application of scenario planning, a tool developed in the high-stakes, uncertainty-ridden world of corporate strategy, for the equally high-stakes and uncertain world of the governance of emerging technologies. The scenario planning methodology is non-predictive, looking instead at a spread of plausible futures which diverge in their implications for different communities’ needs, cares, and desires.

**Results:**

In this article we share how the scenario development process can further understandings of the complex and dynamic systems which generate and shape new biomedical technologies and provide opportunities to re-examine and re-think questions of governance, ethics and values. We detail the results of a year-long scenario planning study that engaged experts from the biological sciences, bioethics, social sciences, law, policy, private industry, and civic organizations to articulate alternative futures of human genome editing.

**Conclusions:**

Through sharing and critiquing our methodological approach and results of this study, we advance understandings of anticipatory methods deployed in bioethics, demonstrating how this approach provides unique insights and helps to derive better research questions and policy strategies.

**Supplementary Information:**

The online version contains supplementary material available at 10.1186/s12910-023-00951-8.

## Background

Recent years have seen rapid advancements in the power, precision, and ease of use of genome editing technologies, rendering research and therapeutic applications in humans increasingly feasible and plausible. These developments have generated considerable apprehension and discussion regarding the potential social, economic, and political implications of human genome editing [1–3]. In embryos, CRISPR is used to study genetics in early human development [4]. In somatic ex vivo approaches, editing platforms are used to deliver gene therapy into animals, revert genetic defects (e.g., hemophilia A) in stem cells, and functionally correct mutations implicated in human Duchenne’s muscular dystrophy [5–7]. Notably, experiments have tested CRISPR-based germline editing to intervene in heritable disease in non-viable human embryos [8–10] and, as widely reported, former Southern University of Science and Technology researcher He Jiankui claimed in late 2018 to have modified CCR5 genes in two embryos brought to term [11]. Nevertheless, numerous technical challenges, including genetic mosaicism, chromosomal rearrangements, and off-target effects, currently hinder the widespread clinical application of human genome editing [HGE] [12]. Indeed, data from He’s unethical human experiment suggests that he inadvertently introduced a different mutation than the one he intended.

However, the risks associated with unintended biological outcomes for patients are not the sole concerns related to HGE, nor are cures for patients the only aspirations. Genome editing technologies are deeply intertwined with complex webs of political, social, and economic relations. Depending on their development and implementation, these technologies could either enhance or undermine social and economic equity, the economic prosperity and military status of various communities and nations, as well as human dignity and social solidarity, among other potential public, private, and political considerations. If such technologies are to produce broad benefits and avoid broad harms, it is crucial to assess their social, political, economic, and ethical potentials and implications. Prospective assessment of technological uncertainties, coupled with values-based inquiries that link social benefit, governance, and desirability of outcomes, can better equip researchers, practitioners, policymakers, and publics to steer the development of HGE towards positive societal outcomes and away from detrimental ones.

Acknowledging this necessity, the field of bioethics and policy discourse surrounding HGE has witnessed an accelerated pace of engagement over the last five years [13]. High-profile expert statements [14–16] and major consensus reports [1, 2, 17, 18] have stressed the importance of forward-looking policy development and inclusive public engagement to guide the future of HGE.

Nevertheless, despite the recognition of the need for forward-looking governance of genome editing by scientific, policy, and ethics communities, the understanding of how to achieve such governance has been limited thus far. This limitation can be attributed, in part, to the inherent uncertainty when attempting to govern emerging technologies. Four decades ago, David Collingridge [19] observed that it is difficult to predict the outcomes of technological innovation early in development, and difficult to alter later-stage “locked-in” technological systems in response to undesirable outcomes. Prior attempts to address this “dilemma of social control” have tended to be institutionally marginal, temporally reactionary, and substantively elite-driven [20–22]. More inclusive and more systemic attempts at governance need to, according to the National Academics of Sciences, Engineering and Medicine (NASEM) report, “deal with both facts and values and in particular how anticipated changes will affect the things people value.” (p. 245).

In response to these challenges, this study aims to address the call for forward-looking, publicly engaged policy development by adopting an anticipatory governance approach. Anticipatory governance employs a range of methods designed to foster foresight, reflection, and flexibility among decision-makers and publics involved with emerging technologies. Upstream efforts in engagement and anticipatory knowledge generation aim to identify relevant values; the ways in which different development and implementation trajectories could support or undercut such; and ways in which researchers, policymakers, and other stakeholders may promote desirable over undesirable development pathways. These strategies are designed to nudge the trajectory of new technologies before they reach a refractory stage of development.

Our study is grounded in theoretical frameworks native to science and technology studies, particularly drawing on anticipatory governance and co-production to inform our methodology and analysis. By co-production we refer to the mutual shaping of science, technology, and society, emphasizing the interdependencies and interactions between scientific knowledge, technological artifacts, and social processes. This positioning means that looking to the future of HGE is not merely a question of technology, but also of social values, power dynamics, economic interests, and cultural contexts. By embedding our research within established theoretical frameworks, we aim to enrich the foundations of our study and contribute to a deeper understanding about how to better investigate the implications of emerging technology.

Initially developed to address societal, ethical, and environmental issues in nanotechnology research, anticipatory governance explicitly focuses on inclusive, value-focused public engagement and future-oriented reflection on the interplay between scientific, technical, and societal change [23, 24]. In the latter vein, anticipatory governance draws upon scenario planning, a foresight method that has long been used by militaries, corporations, and government agencies for purposes of strategic management in uncertain, complex, and volatile operating environments [25–27]. In the context of anticipatory governance, scenario planning is not intended to be predictive, but serves as a tool for critical reflection upon and articulation of the systemic contexts, value tensions, and important potentialities of emerging technologies. Scenario planning works to articulate plausible, challenging, and relevant portraits of what might happen, whether or not they are found desirable [26, 27]. As a research approach, scenario planning involves a rigorous multi-step process that is leveraged to create new knowledge about the future of an emerging technology.

Bioethics has long recognized the importance of “forward-looking” analysis to anticipate emerging issues presented by new technologies, which seeks to prospectively identify ethical challenges so as to minimize potential harms associated with such technologies’ development and use [28]. Yet past efforts at such “proactive ethics” for emerging technologies have been met by a variety of criticisms, including that they lack sufficient scientific grounding, overemphasize regulation to the exclusion of examining deeper questions about desired ends, and fail to sufficiently engage with broader stakeholders and members of the public (and tend to do so too late in the process) [28–31].

Assessing and responding to the ethical and policy challenges presented by the rise of genome editing technologies requires recognition of the numerous and diverse complexities and uncertainties inherent in the scientific process. Assessing the proper aims and scope of these technologies thus requires systematically examining risks and opportunities particular to the scientific features of the technology in question [32]. Yet prior bioethics approaches to anticipate social and ethical issues with emerging technologies have been criticized for too often giving “short shrift” (or even “complete inattention”) to the feasibility of technologies when assessing their ethical implications [33], propagating assumptions about plausibility, safety, or efficacy in the absence of supportive evidence [28]. Scholars have argued that approaches that give insufficient attention to feasibility risk stymieing potentially beneficial research out of fear of “science-fiction scenarios that have little likelihood of materializing” [34]. While it may not be bioethicists’ exclusive role to assess technological feasibility, there is a balance to strike in paying attention to future contexts of use that may be different from contemporary ones.

Secondly, prior bioethical approaches related to emerging technologies have too often emphasized regulatory strategies, while failing to examine deeper questions about what ends we should aim to achieve, and on the related questions regarding the opportunity costs of investing in certain technologies over emphasizing other priorities. By “regulatory strategies” we refer to formal policy and legal instruments, but also more informal mechanisms like ethical codes of conduct or guidelines offered by professional societies. Consequently, such approaches can reinforce technological determinism and the values associated with technological development, to the exclusion of examining which goals we should be pursuing, and why [28, 35]. As noted by Ari Schick, by framing the question of future uses of technologies as “what will we do with the technologies we have,” bioethics has “elide[d] the issue of what technologies we should have and why” [36]. As Schick further explains, by focusing on regulating the future we risk failing to critically examine “the constellation of current decisions, prioritizations, and promises we face today,” and the role of those current decisions in shaping future possibilities [36].

A third critique of prior bioethics approaches in the HGE space is that they fail to sufficiently engage with broad stakeholders, and, when they do engage, they often do so too late in the process, instead relying on governance systems that concentrate ethical authority in the hands of a small number of experts, rather than socially inclusive processes that foster consideration of a broader set of values [20, 37, 38]. While major consensus reports and other high-profile expert statements on the future of HGE have emphasized that robust stakeholder and public engagement should guide policy decisions [1, 2, 11, 12, 14, 15], these statements have generally offered limited guidance on the form such engagement should take. It is perhaps not surprising then that prior engagement efforts have been criticized for occurring too late in the process, once path dependencies have already become established, and for insufficiently capturing the perspectives of the full range of stakeholders, especially those who have been underrepresented in traditional policy-making processes [39, 40].

In what follows, we describe an anticipatory governance approach to scenario development that engaged a broad array of experts and stakeholders from the biological sciences, bioethics, social sciences, law, policy, private industry, and civic organizations through individual qualitative interviews and structured deliberations. We provide a detailed account of the methods deployed, explaining the approach as a sequential, yet iterative, research protocol. We then analyze the strengths and limitations of this methodological approach, analyzing attributes and trade-offs endemic to the approach and its application. Lastly, we suggest that this suite of anticipatory governance tools is well-suited to critically examine the complex and dynamic systems which generate and shape new technologies and may serve as a reproducible model for bioethics scholarship to inform governance for other emerging biotechnologies.

In doing so, we explore how and why these methodological approaches to governing the future of HGE might be valuable to the bioethics community. We are concerned about the limitations of current approaches in bioethics to governing biomedical technologies, especially when there are high levels of uncertainty, ambiguity, and novelty. We propose that scenario planning can facilitate the identification of the social, ethical, and political driving forces and critical uncertainties behind HGE research and synthesize them into a set of plausible future stories to guide deliberation regarding appropriate governance approaches for these new technologies.

## Methods

A set of scenarios about alternative futures of HGE were built through an iterative process that involved conducting key stakeholder interviews and faciliating a two-day deliberation with experts who we engaged in a series of discussions designed to elicit perspectives on dynamic changes in the field, guided by protocols native to scenario planning. Scenarios are “stories describing different but equally plausible futures that are developed using methods that systematically gather perceptions about certainties and uncertainties” [27]. The generation of scenarios relied on interactive group dialogue-- akin to extended focus groups-- involving a careful march through a series of facilitated reflections and elicitations that can be conceived of as a “strategic conversation” [41] or “joint inquiry” [42]. The individuals involved in the deliberation drew on their expertise and experience, which once articulated was then assessed by others and encapsulated in the groups’ outputs. The scientific rigor of the method of scenario planning depends on the drawing in of diverse expertise in a structured and systematic way. In what follows, we share our study protocols, explaining the steps in the method, highlighting the generative nature of data collection, and showing how each deliberative session built on the results of prior sessions, thus accumulating findings and synthesizing the diverse perspectives involved.

### Interview inputs

The scenario planning method involved two main phases: individual expert interviews and a deliberative workshop. We conducted semi-structured interviews with 30 experts across the biological sciences, bioethics, social sciences, law, policy, private industry, and civic organizations in the United States and Western Europe. We selected this set to achieve a diversity of expert perspectives on HGE, thus integrating knowledge on different aspects of the technology and its sociotechnical context [43]. Interviews lasted an hour and were conducted over phone or via the video conference platform Zoom. In each interview, two team members spoke with a single expert, using the interview protocol [see Appendix 1] to probe expert knowledge and insights on the past, present, and potential futures [41] of HGE. With interviewees’ verbal and written consent, we digitally recorded the interviews and had them professionally transcribed. The project team qualitatively coded expert comments using modified grounded theory [44], relating codes to the STEEP framework (social, technological, economic, environmental, and political aspects). Codes were validated by independent triple coding.

### Background material for deliberative workshop participants

Based on a literature review that mapped key gaps in the literature related to the anticipatory governance of HGE [45] and coding of the expert interviews, the research team identified key contextual forces which could shape the future of HGE and constructed a deck of fifty-two “Driver Cards” briefly articulating these forces. The card deck was sent digitally and physically to workshop participants in advance of the workshop. Participants were invited to select for discussion the drivers they found “the most surprising, most intriguing, most dangerous, most contentious, or most hopeful” before the workshop.

A pre-workshop briefing document was also sent to participants digitally and physically, which summarized high profile expert statements [14–16] and major consensus reports [1, 2, 17, 18] stressing the importance of forward-looking policy development and deliberative public engagement to guide the future of HGE. The background materials explained scenario planning as a method within the broader framework of anticipatory governance that can be used to build capacity for foresight, reflection, and flexibility in decision and policy making [24, 46, 47]. In addition, a technical primer to HGE was also offered to ensure a requisite technological literacy across workshop participants.

### Workshop design

The scenario development workshop followed a variant of the “intuitive logics” approach, initially developed by the Royal Dutch Shell Corporation in the 1960s [48] and refined through practice over the last six decades [42, 49, 50]. This two-day facilitated workshop involved structured exchanges of perspectives among study participants and was facilitated by two members of the research team who have expertise in scenario planning methodologies. Due to COVID-19 travel restrictions, we adapted our workshop design for virtual engagement and employed Zoom video conferences and breakout rooms to maximize interactivity, balancing small group discussion and large group, consensus-based deliberations. All group dialogues were guided by specific prompts and were either audio recorded or documented by participants in a virtual whiteboard called Mural. Table 1 shows a summary of the agenda for both days of the workshop and further details are outlined below.

**Table 1:** Process Steps for Scenario Development Workshop

Day 1	Day 2
**Visioning**: Plato’s Cave **Evaluation of Drivers**: Assessing Driving Forces Cards Breakout Debrief from Session Prioritizing Drivers Mapping Uncertainty Breakout Session **Building and Testing a Matrix**: Manufacturing Our Matrix	**Building and Testing a Matrix Continued**: Confirming Scenario Matrix Defining Scenario End States Scenario Logics: Dynamics of Change **Narrating Stories**: Building Scenario Timelines Breakout Session Crafting Scenario Narratives Breakout Session Presenting Scenarios Reviewing the Scenario Set

### Scenario development protocol

#### Visioning

To initiate the workshop and promote engagement, an icebreaker discussion was conducted. We asked participants to design an ideal state for the future of HGE in 2040. This exercise aimed to generate conversation around the distinction between an ideal future, and an uncertain, unpredictable one. The objective was to establish a shared understanding of the ontological stance of the deliberations and the goal of creating plausible and descriptive scenarios, rather than desirable or probable futures [26].

#### Evaluation of drivers

Drawing from insights participants developed by reviewing the Drivers of Change cards in advance of the workshop, participants joined a virtual breakout room to discuss their assessment of the drivers and nominate additional drivers for deliberation. The facilitator added the new drivers to a shared online workspace where all participants could see the composite list and, by voting, indicate the drivers they felt were the most uncertain and most significant for the future of HGE. The facilitator led participants to rank the most important and most uncertain drivers for the future of HGE following the nominal group technique [51]. In the type of complex socio-technical systems within which scenario planning deals, there are always more potential drivers of outcomes than can be treated in a single exercise or set of scenarios. Ranking of drivers by importance and uncertainty allows the scenario development process to focus on a subset of drivers which could conceivably produce a highly divergent spread of possible futures, which in turn facilitates robust attention on a sufficiently wide variety of potential developments. In this move, participants evaluated not only which drivers mattered most for the evolution of HGE, but also which ones were most shrouded in unknowns.

#### Building and testing a matrix

The next phase involved crafting scenarios based on the top critical uncertainties ranked by the workshop participants. The facilitators proposed various 2 × 2 matrices that intersected two independent drivers with one another [52, 53]. The 2 × 2 matrix is used in scenario planning to scaffold a foundation across two critical uncertainties to produce a diverse spread of futures across which other important uncertainties and drivers can be explored divergently [50, 54]. The workshop participants then debated which combination would yield the most dynamic interactions between intersecting extremes of the critical uncertainties [50].

#### Narrating stories

Informed by driver discussions and the contours of the 2 × 2 matrix, participants were asked to expand upon scenarios falling at intersecting extremes of two variables: (1) the distribution of access and power and (2) the degree to which private or public values guide HGE development. In order to flesh out the scenarios, participants were asked to creatively imagine possible futures for HGE within each of the four quadrants. Then, participants built out these future state scenarios by constructing ten-year timelines of events across social, technological, environmental, economic, and political dimensions of HGE, creating media headlines across each temporal landscape (the year 2020, 2030, and 2040). Media headlines offer a concise, newsworthy focus of attention to encourage reasoned speculation about how a particular future might unfold in more concrete terms. The timeline also ensures that there is a logical causation underlying the scenario worldbuilding process.

Once the structural elements of each scenario were established by the expert group, including logical sequence of events and treatment of social, political, environmental, economic, and technological dimensions, storytelling was employed to further develop and refine each scenario. Describing in detail what the future state might look like involved integrating the other uncertainties not selected as a primary structuring pair. Narrative storytelling is a powerful tool to cinch together disparate elements and provide a communicative anchor to help articulate new prospective realities. Information is more easily remembered when presented as a story and the narration process of constructing a scenario serves to bring the future world to life in a way that simply recounting possible issues does not [55].

Following the workshop, the research team compiled and consolidated the inputs into scenario narratives [see Appendix 2]. In this polishing process, the research team paid special attention to ensure fidelity to the workshop conversations to ensure that the perspectives of the participants were valued and validated. Key themes were compared across the scenarios to ensure divergence, and tensions were amplified across and between the scenarios to create dynamism. The research team went through several rounds of iteration, including feedback sessions with an expert panel and a sub-set of workshop participants.

## Results

The workshop resulted in the development of four scenarios, crafted by the participating experts informed by their own knowledge and supplemented by literature review and the qualitative interviews. These scenarios presented different stories about the future socio-political contexts of HGE. This suite of methods produced different future states that honed in on a limited number of critical uncertainties, and reflected emergent social, technological, economic, environmental, and political issues that were determined to have pivotal effects on the shape of the future of HGE. The research protocol yielded thick descriptions of important drivers of change, along with estimations of their potential and divergent outcomes. In this next section, we elaborate upon these key uncertainties revealed through the workshop, as well as the resulting scenarios.

One key uncertainty surfaced by participants was how the proliferation of genome editing technologies leads to new actors. CRISPR-cas9-based genome editing tools have achieved wide international uptake across many life-science communities, enabling nontraditional research actors, including small entrepreneurs and self-identified biohackers, to readily access certain CRISPR complexes. The extent to which preexisting broad proliferation will diversify actors in genome editing spaces or complicate efforts to surveil or regulate use remains to be seen. Group conversations highlighted key uncertainties underpinning variation in the proliferation of HGE, including how the more variation there is in actors engaging with these technologies, the more difficult they will be to regulate, control, and track.

Another key set of uncertainties that animated the scenarios related to the issues of social engineering, the threat of eugenics, and population control. Recent years have seen resurgences in ethnonationalism and the politics of racial superiority, alongside longstanding discourse of over- or under-population on scales of the human species or subgroups. It is unclear how and to what extent eugenic and ethnonationalist currents in contemporary political discourse might interact with the development of HGE. Group conversations highlighted key uncertainties like the extent to which genome editing is shaped by state vs. individual power, cultural norms around uniformity vs. diversity, or optimization vs. diversification, and wealthy elites vs. totalitarian regimes.

Unauthorized or rogue actors was another persistent topic of discussion. One person’s rogue actor is another person’s hero. Therefore, who makes the rules determines what practices and conduct will be considered outside of the normative rules for HGE innovation. Increasing involvement of citizen scientists, biohackers, and non-institutional players in biotech hubs, connected to research universities and other loci or expertise, in the US and abroad, might impact the evolution of biomedical technologies. Group conversations highlighted key uncertainties about future evolutions in centralized vs. fragmented power, and who determines who is rogue and who isn’t, as well as what role financial power plays in convention setting.

Experts discussed the role of competition and the rhetoric of inevitability around HGE, or the idea that no one can afford to fall behind. Individual investigators or research teams may feel similar pressures or incentives to push forward with applications. Future regimes of consolidation versus distribution of power of political players in this space are highly uncertain. Group conversations highlighted uncertainties around how questions of human rights may fold into the development of these technologies and whose morality will be used to decide future trajectories and priorities in HGE.

Some workshop participants focused on the role of the military and suggested that HGE could be used to provide improved immunity, stamina, or other enhancements to soldiers, providing a fraught and dire incentive for development. Military actors, if interested, could direct immense resources and pressure toward genome editing development. Group conversation highlighted uncertainties around how the geopolitics, number of power centers, and levels of hostility will greatly impact the use and development of HGE by militaries.

**Fig. 1:**
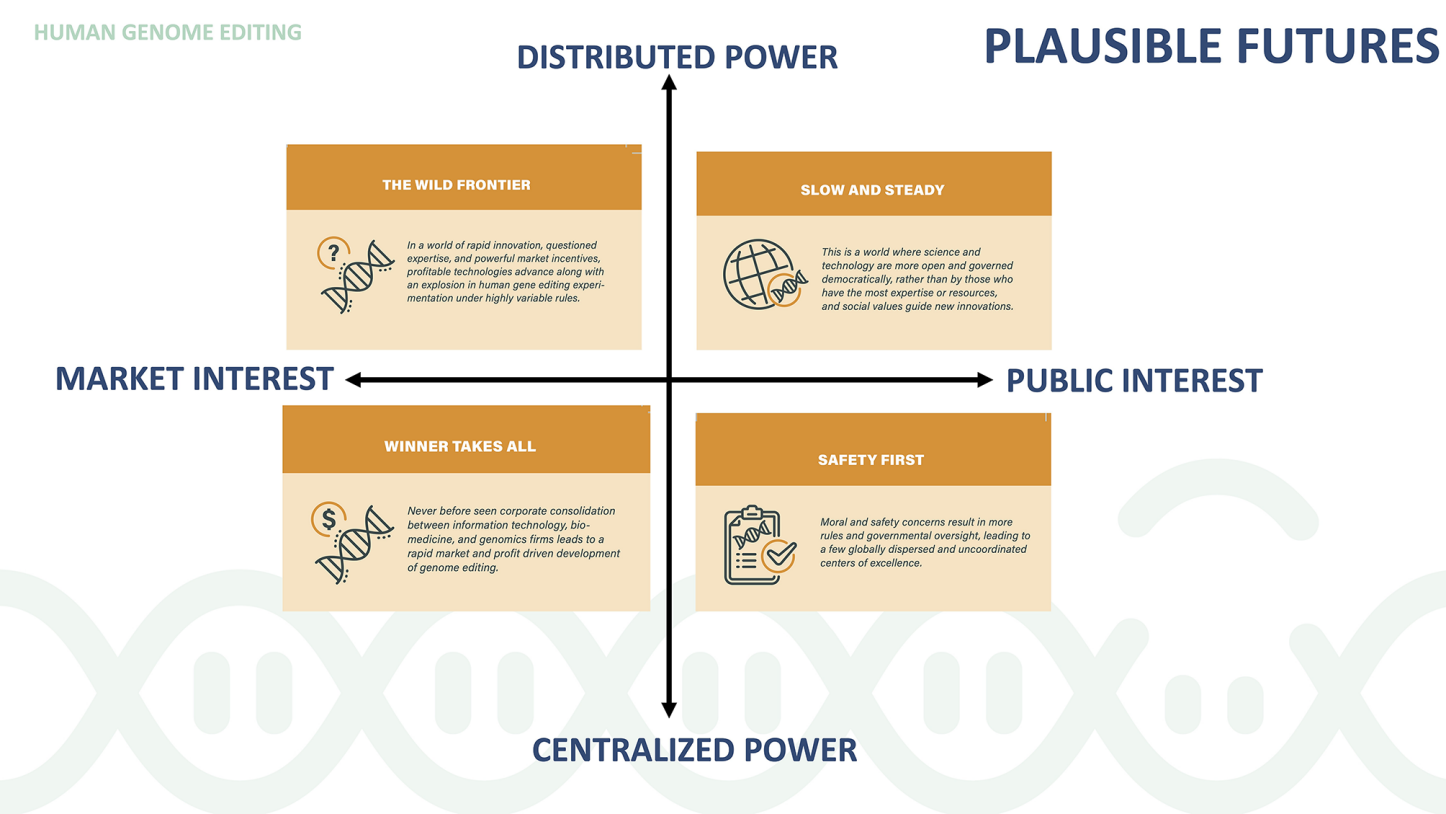
2 X 2 Matrix of Plausible Scenarios for 2040

These many uncertainties were assessed, debated, and resolved into a scenario matrix across an axis focusing on two main factors: “distributed power versus consolidated power” and “driven by private interests versus driven by public interests”. Beyond those two structuring uncertainties, each of the scenario stories explores causal relationships between several tensions already evident today but expected to evolve differently in the future (see Appendix 3 for a comparison of the scenarios across key factors). The workshop participants named the scenarios: Wild Frontier, Slow and Steady, Safety First, and Winner Take All (Fig. 1- “Scenario Matrix”).

The first scenario investigated a world of rapid innovation, contested expertise, deregulation, and powerful market incentives. **The Wild Frontier** scenario imagines how profitable technological development proceeds in tandem with an explosion in risky HGE experimentation under highly variable rules. What counts as “science” and “knowledge” is widely contested, as are public views on HGE. Public knowledge of the mechanics or realistic limits of HGE is minimal, and perception is shaped largely by marketing. Access is widespread, though there is little guarantee that HGE is real or effective. The most powerful and effective treatments are exorbitantly expensive. Formal governance of HGE is largely national and sometimes even local, though economic elites command significant informal authority. Oversight is spotty and local.

In a turn away from elite science serving the few, the **Slow and Steady** scenario envisions a world where science and technology are more open and governed democratically, and social values steer new innovations. Public understanding of science in general-- and HGE in particular-- are on a steady rise as a result of broad, systematic, and productive public engagement with experts and stakeholders in academia, industry and government. However, cracks appear when rogue actors, unhappy about the status quo, use disinformation to sow confusion. A moratorium on germline editing, “a War on Monogenic Disease,” and consensus criteria for approving gene therapies serve as organizing principles for formal cross-national governance. Global application of the “collective good” principle in coordinated cross-national collaboration ensures equitable access to HGE technologies in the broadest possible scale to all compliant actors in government, industry, and academia. Application areas are matched with the highest public needs at the level of nation states, as well as at the level of genetic diseases affecting the most vulnerable populations.

In the **Safety First** scenario, safety and moral concerns give rise to increased regulation and governmental controls, leading to a coordinated global patchwork of centers of excellence. Widespread fears about messing with nature breed an era of caution in the biomedical sciences. After numerous catastrophes arising from the relatively unregulated use of CRISPR and an explosion of in-vitro experimentation world-wide, consensus is reached that heavy regulation is the only way forward. Whether due to religious concerns or anxieties about downstream risks, a massive public backlash forces HGE advances out of the limelight. While there are no global systems of governance, countries form confederations of governance regimes to align resources around shared goals and priorities and vary depending upon a country’s permissibility of HGE. Access to HGE is limited to those with conditions that align with their country’s prioritized applications of HGE technology or with the financial means and ability to travel to where particular HGE uses are permissible.

In the **Winner Takes All** scenario, unprecedented corporate consolidation between IT, biomedicine, and genomics firms leads to a rapid development of HGE, but only for the global elite. This new world obsessed with optimization through technology emerges following the tech-lash of the early 20s, where initial public outcry leads large IT corporations to step up and take on more responsibility for their innovation and business practices. Tech giants succeed in internalizing social responsibility and create so many new jobs with a bio-boom that they are allowed to self-regulate. With the limits of their growth unchained, tech companies increasingly move into new domains, providing solutions to improve health care, ease poverty, fight crime, and mitigate global climate change. Due to a lack of access or inclusive governance mechanisms, the public has limited interest in and understanding of scientific enterprise. HGE is understood mainly as a tool of the rich and the majority of the globe only knows about HGE via social media and popular news outlets, which focus on extremes in enhancement and legal debates among wealthy entrepreneurs. Governance is largely left to multinational corporations with limited public oversight.

The scenarios summarized here are not designed as predictive tools, but exploratory and illustrative ones that aim to add complexity and a deeper exploration of systemic dynamics to ethical debate. It is not possible to know and prepare for all the features of a single possible future, nor is that the aim of this approach. Instead, the research goal is to articulate and review an array of important possibilities in order to support preparations that can support resiliency and effective governance across a wide range of plausible futures. In this research case, the scenarios were then used to frame public deliberations to explore public values. The scenarios serve to reveal previously unseen trends, potential dangers and opportunities, surprising relationships, and points of leverage by which actors can work to promote desirable outcomes and mitigate undesirable ones.

## Discussion

The scenario planning methodology employed in this study generated four sceanrios that model different logics of change, offering diverse expert perspectives on future socio-political contexts of HGE. Intermingling a number of diverse variables to create divergent vantage points has the advantage of enabling more reflexive views on questions of values and governance. As a research method, scenario building has many positive attributes, or affordances, that generate useful data and fresh insight. In this section, we describe these affordances and limitations, in order to advance an understanding of the potential value of the methodology for improving bioethical reflection.

We can see that a key affordance of the methodology is the way in which it encourages reflection on the evolution of socio-technical change. Rather than taking for granted particular technological trajectories or the durability of certain regulatory regimes, the approach fundamentally asks, “what if things were different?” Through dialogue and debate that opens up reflection into the fundamental motors of change, the method challenges assumptions and broadens conceptual categories that might otherwise lock-in thinking and ethical reflection.

One of the mechanisms through which this opening up occurs is through systems thinking that gives equal footing to social values, economic pressures, and regulatory efforts along with technological trajectories. In this way, the approach embraces the Science, Technology and Society (STS) invitation to include broader understandings of the diverse array of factors that impinge upon the development of an emerging technology and the social organizations that produce such knowledge. In this way, positive (or detrimental) societal outcomes are not a mere function of the technical performance of a technology but are linked to how that technology is embedded in a wide variety of socio-political systems and economic configurations. An assessment of the ethics of an emerging technology must take into account systems dynamics and effects or else risk neglecting how a diverse set of norms, institutional structures, and incentives shape outcomes. The methodology’s use of the STEEP framework to articulate the social, technological, environmental, economic, and political drivers corrects a tendency within bioethics to ignore how dynamic and complex factors influence outcomes by relying too heavily on technological determinism [28].

Another affordance of the approach links to how such systemic socio-technical interactions are investigated through storytelling. By crafting future-oriented stories, workshop participants were invited to integrate diverse factors into vividly represented new worlds. Narrative is well understood as an integrating method [27, 56] and functions in this context as a way to explore how diverse factors might evolve to constrain or enable others. By colliding change dynamics—for instance, how a social movement against expertise might interact with wider accessibility of HGE tools—storytelling clarifies causal relationships at play. Again, instead of merely extrapolating along one variable to produce an alternative future, scenario planning mixes different variables to explore the dynamics between them to better understand how the factors might influence one another.

The method also benefits from substantive engagement with a wide variety of experts. Such engagement with experts also adds to grounding scenarios in plausibility [31]. In our study, we involved a diverse array of disciplinary and stakeholder perspectives and sought to level the playing field where each were given equal weighting. This has the effect of offering a type of “extended peer review” on HGE which becomes essential when “facts [are] uncertain, values in dispute, stakes high and decisions urgent” [57]. While such interdisciplinary inclusion is necessary to rally the requisite expertise needed for a more systems-based inquiry, the methodology also serves to create bridges between the different perspectives. It is well known that each discipline and stakeholder group maintains its own foci, intellectual histories, problem framings, and sites of contestation. These are too often kept siloed and insular. Discussion of HGE has tended, even within a single report [1] to neatly segregate technical capabilities and potentials, clinical-ethics considerations, and rather fuzzily articulated societal possibilities. Roughly put, ethicists discuss clinical ethics and ethical issues related to research and application, technicians discuss technical problems, and social scientists critique the forms of authority and values guiding the genome editing development. Scenario building workshops can create a space where different disciplinary and stakeholder perspectives can confront and challenge, meld together, and become more productively engaged with one another.

Together, these affordances add up to a more rigorous, more systematic approach to explore the ethics of a socio-technical system that is emerging, as yet unclear, and riddled with uncertainty. The scenario methodology provides structure and accountability to test assumptions, open up taken-for-granted categories, and dissolve a linear approach to extrapolating singular variables. It deploys a suite of different time-tested social science research methodologies and in doing so, ensures rigor through iteration, where each phase builds upon and verifies the results from the last-deployed method. The quality of the data generated is persistently validated through interviews, dialogue, ranking, storytelling, and vetting with others.

Though there is much promise in approaching anticipatory governance through scenario planning methodologies, there are some limitations to the approach. In some cases, there is a Janus head quality to the method. For instance, one of the strengths of the approach is how it bases results on the perspectives of a wide variety of stakeholders. On the flip side, data quality is constrained by who is involved. This means that the quality and diversity of those involved is critical to the outcomes produced. The methodology is thus susceptible to failure if the right constellation of actors is not involved. Who is involved is not a trivial matter. For our research project, we generated a list of desired disciplines and perspectives based on a rigorous literature review to map the key issues, controversies, and ethical dilemmas wrapped up in HGE [45]. We paid careful attention to securing high caliber participants based on their scholarly contributions, status and stake in the community, and type and breadth of expertise. But how lines are drawn around the community—and whose point of view is thus weighted— is subjective, even with the best of checks and balances. We are at a point of reckoning with issues of justice, diversity, and inclusion, and grappling with which voices are excluded from seats at the table and so these questions are not trivial. Further, we note that the ability to participate, including the time to devote several days to such an effort, is a privilege that many cannot afford. Thus, the selection of experts can have the tendency to reify existing power inequalities and yield results that simply reinforce the status quo. A critical success factor in ensuring good data quality is to ensure just representation, a breadth of relevant expertise, and prioritizing participation of voices too often discounted.^1^

Another weakness in the approach relates to its sensitivity to good facilitation. With the main methodological intervention being a dialogue-based workshop following a precise architecture of conversation, skill in facilitation is paramount. A good facilitator will credibly explain the purpose and operations of the method, ensure steady progress through a complicated set of discussions, work to include all participants, and anticipate the hurdles typically encountered and have correctives on the ready. Navigating through the process fairly required steady facilitation to allow the debate to unfold without losing sight of the need to make progress and the overarching goal of the research to promote reflection on future governance of HGE. Such skills are developed over years of practice and require several interpersonal capacities in addition to know-how of the techniques.

A last limitation of the method, that is also double-edged, relates to how challenging it is for academics and experts to speculate. On the one hand, scenario planning provides a stepwise scaffolding to support an incremental building up of expansive, future-focused points of view. With each stage in the process, anticipatory knowledge is crafted and vetted, formulating the building blocks of a scenario set that are then rendered as narratives. But conjecture is nevertheless hard and invites those lauded for knowing things to delve into what they do not—and cannot—know. In our process, we worked to loosen that grip on surety through the pre-workshop brief that explained the methodological approach, by grounding the inquiry in well-researched drivers, and in using icebreakers and other techniques to encourage imagination.

Taken as a whole, with these strengths and limitations, we assert that this methodology, conducted well, can provide a fruitful approach to research into the bioethics of emerging technologies. This suite of methodologies fall prey to some shortfalls of any approach that relies on expert deliberation. Any type of deliberation or qualitative research that involves surfacing perspectives (interviews, surveys, focus groups, ethnography, etc.) is subject to critiques about who is involved, with which interests, and with which capacities for authentic sharing. What’s special about this approach to bioethics’ quest to grapple with emerging biomedical technologies is the generative, imaginative, and iterative nature of the knowledge produced. This protocol is not just about extraction or articulation but also about live generation and co-construction of results that co-creates new knowledge and understandings.

## Conclusion

We have demonstrated the contributions scenario planning methods can make in enabling experts and stakeholders to identify, synthesize, and assess potential future states of emerging biomedical technologies. As a research methodology, scenario planning relies on specialized interview techniques that nurture reflexivity, interdisciplinary generative dialogue drawing on systems modeling, and creative storytelling that clarifies causally linked cascading effects. By surfacing the key uncertainties that can shape the future paths of HGE and encouraging a deeper reflection on the desired ends for the technology and the ways in which different approaches could support or undercut those ends, this method can support the ability of researchers, policymakers, and other stakeholders to identify governance approaches that may better realize those ends.

As we noted at the outset, bioethical reflection on emerging technologies has faced criticism for being mired by weak empirical grounding and focusing attention on hyperbolic or implausible concerns. The scenario planning approach, through substantive and structured engagement with a wide variety of experts, can better ground ethical reflection in plausibility [26, 31] enabling prospective analyses.

We also argued that too often bioethical reflection is overly focused on regulatory issues locked into current understandings of technological feasibility, embracing a techno-centric perspective which fails to sufficiently engage with the broader set of relevant ethical questions, including, importantly, to what ends we should aim to achieve. The scenario planning methodology opens up the scope, encouraging reflection on the evolution of socio-technical change and the role of social values, economic pressures, and regulatory efforts to shape technological trajectories, rather than taking those trajectories as a given—offering opportunities to move beyond a reactionary approach.

The third shortcoming of some bioethical reflection is that prior approaches have tended to be too narrow and siloed. The scenario planning methodology affords substantive interdisciplinary engagement which encourages reflection on a broader set of values. The approach offers advantages both in moving beyond siloed approaches, while also offering opportunity to develop groundwork for downstream public deliberation activities that better frame a broader set of values and trade-offs.

As HGE is but one biomedical innovation among many underway, navigating uncertainty and working to ensure good governance decisions under novel conditions will continue to be a 21st century necessity in responsibly steering innovation. Uncertainty, coupled with acceleration and novelty, creates challenging circumstances for the array of actors—from bioethicists, to scientists, publics, entrepreneurs, and regulators—to make good choices that yield positive societal outcomes. Anticipatory governance methods provide a disciplined approach for bringing together diverse voices to engage purposefully in thinking through such complexity and its implications for the longer term. The rigorous and broadly scoped survey of important potentials and drivers afforded by scenario planning supports more integrated, more systematic, and more actionable articulations of important possibilities which serve as a helpful corrective and supplement to conventional bioethical reflection on emerging technologies.

### Electronic supplementary material

Below is the link to the electronic supplementary material.

Supplementary Material 1

## Data Availability

The datasets used and/or analyzed during the current study are available from Christopher Scott on reasonable request.

## Footnotes

1 This particular project benefits from a phase of dedicated, deliberative public engagement to counterbalance the potential expert bias of the scenario development process, developed on the participatory technology assessment method detailed by Kaplan and colleagues [58].
