# Supplementary material for: Researching the future: scenarios to explore the future of human genome editing
Source: BMC Med Ethics. 2023 Sep 21;24:72. doi: 10.1186/s12910-023-00951-8 (PMC10512597; doi:10.1186/s12910-023-00951-8)
Supplement: Supplementary file 1 — Supplementary Material 1 [file 12910_2023_951_MOESM1_ESM.docx]

**Researching the Future: Scenarios to Explore the Future of Gene Editing**

**APPENDIX**

**APPENDIX 1: Scenario-based Qualitative Interview Protocol**:

**Domain 1: Personal and professional background and relation to human genome editing research**

1. To start, could you briefly tell me about your role and your personal or professional relationship to human somatic or germline genome editing research?
2. In your view, is human genome editing a priority for biomedical science and human health? Why or why not?

**Domain 2: Anticipatory states**

1. What have been the pivotal events and developments in human genome editing? Are there any events over this history that have surprised you?
2. What plausible advances in science and medical care might we see from human genome editing in 10 years? 20 years?
3. Which variables do you see as likely to be the most influential in shaping the future of human genome editing?
4. What factors do you think aren’t on people’s radars that should be? What variables have current debates failed to adequately consider?
5. Which stakeholders do you see as most influential in decision-making about these technologies? How, if at all, do you envision this changing in the future?
6. What are the key social, ethical, legal, economic or policy questions that must be resolved?
7. If you were a benevolent dictator, what action would you take to improve the field of human genome editing for the future?

**Domain 3: Open ended**

1. What should be considering regarding the governance and future use of these technologies?
2. If we were to speak to two experts or other thought leaders about this issue, who should we talk to, and what should we make sure to ask them?

**APPENDIX 2: Scenario Narratives Developed in Workshop**

The first scenario investigated a world of rapid innovation, contested expertise, deregulation, and powerful market incentives. **The Wild Frontier** scenario imagines profitable technological development proceeds in tandem with an explosion in risky gene editing experimentation under highly variable rules. This scenario depicts a world of fast technology and unfettered scientific creativity, challenged by deep divides in access to commercialized health care and the erosion of faith in orthodox science. In a laissez-faire environment with limited controls, everyone is a player. Due to proliferation of variant techniques and widespread adoption, many biomedical companies have been unable to maintain monopolies on genome editing platform technologies. The tools of genome editing are broadly available, leading to widespread laboratory and human experimentation with little oversight.

Unregulated genome editing clinics proliferate. Smaller medical providers pick up skills and tools to offer genome editing services—some genuine, most ineffective at best—to mid-income customers. Assisted reproduction clinics, historically under regulated, include genome editing in libraries of cost-raising “add-ons.” Firms with real HGE capabilities focus on serving wealthy “medical tourists”, the only ones willing and able to pay for and to travel to receive vetted and cutting-edge interventions. Without regulation serving as a quality control, and with trust in government and scientific institutions at an all-time low, the average consumer-citizen has little way to distinguish high quality services from dangerous or ineffective offerings. For consumers in the know, with the right connections, and capital, powerful treatments are available; for everyone else, there’s much risk and uncertainty.

Regulatory agencies fail to keep up with dubious “genome editing” uses while attending to more mainstream responsibilities, including those interventions moving through approved clinical trials. Scientific enthusiasm, support for entrepreneurial “biohacking”, unethical actors, and the drive to deliver products to eager consumers and commercialized health care providers make it difficult for consumers to tell efficacious treatments from snake oil. Under continued increases in nationalist and isolationist sentiment and breakdowns in international relations, international governance frameworks have failed, leaving HGE governance spotty and jurisdictionally fragmented. While some small outfits sprout up claiming to treat niche disease and conditions, they do so without investing in clinical trials and so efficacy and long-term effects are largely unknown. Even without the rigor of old systems of regulation, much experimentation leads to novel inventions and accelerates the use and uptake of new medical technologies.

The second scenario highlighted the social control of science and technology. In a turn away from elite science serving the few, the **Slow and Steady** scenario portrays a world where science and technology are more open and governed democratically, and social values steer new innovations. Driven by the Successful development and delivery of COVID vaccines, momentum formed for a new public-private-philanthropy business model for biomedical innovations and cross-national collaborations. This drew in many new actors and cooperation was found to be more fruitful and economic than competition. Open data sharing began to increase the rate of success in clinical trials for gene therapies, producing close to a dozen therapies with positive clinical data. As technological and economic constraints on data sharing lessened, and new cooperative and intellectual property agreements emerged, the true power of open science was finally unleashed. Aided by machine learning and shared tools, progress in gene editing became rapid.

International agreements on open sharing of data, homogeneity of ethical frameworks for gene editing, and lower barriers to entry led to a vibrant innovation ecosystem worldwide, yet one that is tethered to the public good. Regulatory approval processes are more streamlined, functional and evidence-based yet there are strict rules around ethics and a requirement to create better societal-level outcomes. This added layer of oversight slows the innovation system, but ensures focused research, increases the safety of new applications, and set the stage for high public acceptance of gene editing.

In the **Safety First** scenario, safety and moral concerns give rise to increased regulation and governmental controls, leading to a global patchwork of centers of excellence. Widespread fears about messing with nature breed an era of caution in the biomedical sciences. After numerous catastrophes arising from the relatively unregulated use of CRISPR and an explosion of in-vitro experimentation world-wide, consensus was reached that heavy regulation was the only way forward. Whether due to religious concerns or anxieties about downstream risks, a massive public backlash forces gene editing advances out of the limelight.

Considered a potentially dangerous technology, but also noted for its unprecedented power to offer treatments for stubborn diseases, governments around the world decide to tightly control the development of CRISPR, particularly for germline interventions and/or so-called “enhancements.” Harsh penalties for those that transgress national laws regarding HGE, including steep fines, loss of licenses, and/or imprisonment became common. Public investment continues in earnest, with large amounts of funding devoted to policing the distribution and use of gene editing tools and know-how.

In almost every country, mission-driven research advances health priorities, but it also increases the prominence of politics in allocating funds, determining directions of development, and deciding who can and cannot conduct research. This politicization gives rise to a new style of lobbying whereby patient groups, anti-HGE activists, medical researchers and biotech firms all vie for attention from the labs and the exclusive bodies that govern them. Diseases that affect politically sympathetic populations (e.g. cystic fibrosis) or those with well-resourced and highly mobilized interest groups (e.g. breast cancer) receive a disproportionate share of research resources. By contrast, orphan diseases generally get neglected.

What emerges around the globe is a fragmented ecosystem of discrete islands, or leagues, each focused on different areas of application or disease treatment. Those living in countries where HGE is banned or limited, travel to “pro-HGE” countries to pursue desired treatments and a thriving medical tourism industry develops. Scientists who are keen on developing and conducting research with HGE also migrate to countries where technologies or their specific applications are allowed, creating cadres of so-called R & D Refugees. This piecemeal development is marked by punctuated progress, with long periods of latency followed by rapid acceleration in targeted advancements.

In the **Winner Takes All** scenario, unprecedented corporate consolidation between IT, biomedicine and genomics firms leads to a rapid development of genome editing, but only for the global elite. This new world obsessed with optimizing through technology emerged following the tech-lash of the early 20s, where initial public outcry led large IT corporations to step up and take on more responsibility for their innovation and business practices. Tech giants succeeded in internalizing social responsibility and created so many new jobs with the bio-boom that they were allowed to self-regulate. With the limits of their growth unchained, tech companies increasingly moved into new domains, providing solutions to improve health care, ease poverty, fight crime and mitigate global climate change.

With this new social capital and prowess, the size and scope of tech companies increased, enabling armies of lawyers to rapidly protect their stakes in the human genome. With intellectual property squarely in the hands of few global corporations, genetic editing became a private affair with independent entrepreneurs and academic institutions effectively out of the market. As the technologies became increasingly complex, increasing capital costs of treatment development and delivery mechanisms placed further barriers to entry. Governments are also consumers, contracting with tech giants for these solutions, while still robustly sponsoring HGE research.

A new population of ultra-wealthy, immunologically and otherwise enhanced elite socialize and work together in larger and larger enclaves while those unenhanced populations are increasingly relegated to low paying, high risk jobs. While there are different norms at play with some consumers opting to ‘go natural’, and entire countries effectively left out of the game, medical elites become the barometer of the new normal and those that cannot (or choose not to) play face increasing disadvantages. In some circles, super-ability trumps natural ability, making natural ability the new disability. In other circles, resisting normalization becomes a lifelong quest and political mandate. New social rifts emerge, with some calling genetic fashion as racist, an overreach of surveillance or destroying biological sovereignty; others viewing a failure to modify as child abuse.

As the marketplace strives to supply newer and better products to a smaller and smaller population, fissures in public health emerge. By outsourcing public goods from IT infrastructure to public transportation to tech companies, basics like vaccines and natural prenatal care dwindle. People without immunological “upgrades” are less able to travel, limiting global mobility or adding new risks to those who must travel for work. This leads to further segmentation between those active in shaping the global economy and those at its mercy.

**APPENDIX 3: Scenario Comparison Table**

| **Key Scenario Features** | **Many Actors, market values** | **Many actors, public values** | **Few actors, public values** | **Few actors, market values** |
| --- | --- | --- | --- | --- |
| Scenario | The Wild Frontier | Slow and Steady | Safety First | Winner Takes All |
| Central **Logic/Engine** of the Scenario | Low regulation, uneven outcomes | Open innovation, guided by public values | Early regulation; path dependent tech development | Efficiency & private profit driven; unregulated |
| **Access** to gene editing | Limited with medical tourism & huckerterism | Access is curated but not restricted, open experimentation | Varied, depended on country’s permissibility | Limited by personal wealth |
| Loci of **authority** | Informal | Formal, global | Formal; international coalitions | Informal; dispersed globally across large corporate entities |
| Dominant **application** areas | Athletic and military | Public health with low level of complexity | Few; somatic and only therapeutic | Somatic & germline, enhancement (athletic and aesthetic) |
| # and type of **players** | Many private sector companies | Global public/private philanthropy | Few, research institutions | Few, ultra-wealthy |
| Modes of **power** exerted | Research funding | Regulatory, science + risk assessments | Interest group politicized decision making | Popular demonstration, social media, celebrity influence |
| Most powerful **actors** | Companies and wealthy individuals | Experts / technocrats | Prestigious academic institutions and federal funders | Corporate scientists & high net-worth individuals |
| Dominant **values** driving innovation | Profit, applied science | Purpose, evidence driven | “Mission driven” | Speed, profit, human hubris |
| Major **problems** the scenario foregrounds | Lack of trust & dangerous solutions | Less innovation, but high-quality tech & high level of access | Path dependence; first investments narrow innovation pathways | Severe inequality |
